# Identification and Pyramiding Major QTL Loci for Simultaneously Enhancing Aflatoxin Resistance and Yield Components in Peanut

**DOI:** 10.3390/genes14030625

**Published:** 2023-03-01

**Authors:** Gaorui Jin, Nian Liu, Bolun Yu, Yifei Jiang, Huaiyong Luo, Li Huang, Xiaojing Zhou, Liying Yan, Yanping Kang, Dongxin Huai, Yinbing Ding, Yuning Chen, Xin Wang, Huifang Jiang, Yong Lei, Jinxiong Shen, Boshou Liao

**Affiliations:** 1 Key Laboratory of Biology and Genetic Improvement of Oil Crops, Oil Crops Research Institute of the Chinese Academy of Agricultural Sciences, Ministry of Agriculture and Rural Affairs, Wuhan 430062, China; 2 National Key Laboratory of Crop Genetic Improvement, National Sub-Center of Rapeseed Improvement in Wuhan, Huazhong Agricultural University, Wuhan 430070, China

**Keywords:** peanut, aflatoxin resistance, seed weight, QTL mapping

## Abstract

Peanut is susceptible to *Aspergillus flavus* infection, and the consequent aflatoxin contamination has been recognized as an important risk factor affecting food safety and industry development. Planting peanut varieties with resistance to aflatoxin contamination is regarded as an ideal approach to decrease the risk in food safety, but most of the available resistant varieties have not been extensively used in production because of their low yield potential mostly due to possessing small pods and seeds. Hence, it is highly necessary to integrate resistance to aflatoxin and large seed weight. In this study, an RIL population derived from a cross between Zhonghua 16 with high yield and J 11 with resistance to infection of *A. flavus* and aflatoxin production, was used to identify quantitative trait locus (QTL) for aflatoxin production (AP) resistance and hundred-seed weight (HSW). From combined analysis using a high-density genetic linkage map constructed, 11 QTLs for AP resistance with 4.61–11.42% phenotypic variation explanation (PVE) and six QTLs for HSW with 3.20–28.48% PVE were identified, including three major QTLs for AP resistance (*qAFTA05.1*, *qAFTB05.2* and *qAFTB06.3*) and three for HSW (*qHSWA05*, *qHSWA08* and *qHSWB06*). In addition, *qAFTA05.1*, *qAFTB06.3*, *qHSWA05*, *qHSWA08* and *qHSWB06* were detected in multiple environments. The aflatoxin contents under artificial inoculation were decreased by 34.77–47.67% in those segregated lines harboring *qAFTA05.1*, *qAFTB05.2* and *qAFTB06.3*, while the HSWs were increased by 47.56–49.46 g in other lines harboring *qHSWA05*, *qHSWA08* and *qHSWB06*. Conditional QTL mapping indicated that HSW and percent seed infection index (PSII) had no significant influence on aflatoxin content. Interestingly, the QT 1059 simultaneously harboring alleles of aflatoxin content including *qAFTA05.1* and *qAFTB05.2*, alleles of PSII including *qPSIIB03.1*, *qPSIIB03.2*, and *qPSIIB10* and alleles of HSW including *qHSWA05*, *qHSWB06*, *qHSWA08* had better resistance to *A. flavus* infection and to toxin production and higher yield potential compared with the two parents of the RIL. The above identified major loci for AP resistance and HWS would be helpful for marker-assisted selection in peanut breeding.

## 1. Introduction

Peanut (*Arachis hypogaea* L.), or groundnut, is an important oil and cash crop in the world, widely cultivated in China, India, the United States, Nigeria and other countries. In 2021, the global peanut planting area was 32.72 million ha with a production of 53.93 million tonnes [1]. However, peanut is among the agricultural products conducive to aflatoxin contamination. Aflatoxins are mycotoxins produced in secondary metabolism of fungi belonging to *Aspergillus* section *Flavi*, including *Aspergillus flavus*, *A. parasiticus*, *A. nomius* and *A. pseudoamarii* [2]. *A. flavus* is a common saprophytic aerobic fungus which is known as a main fungal pathogen of corn, legumes and peanut [3,4]. The International Agency for Research on Cancer (IARC) has classified aflatoxins as a Group 1 carcinogen causing negative health effects, including death, in both humans and domestic animals [5,6]. In China, the potential liver cancer risk attributed to aflatoxins exposure from peanuts and peanut oil varies from 0 to 3.43% in different regions, and children aged 2–6 years have the highest aflatoxin exposure level [7].

In general, there are two main mechanisms of peanut seed resistance to aflatoxin contamination: seed infection and aflatoxin production resistance [8]. Transcriptome analysis by RNA-seq indicated that quercetin induced cell death by suppressing the expression of genes related to proliferation and development of *A. flavus*. PR proteins have been shown to be involved in the regulation of plant resistance to aflatoxin contamination. In seeds of resistant genotypes after inoculation with *A. flavus*, β-1,3-glucanase activity was significantly higher in the susceptible genotype [9]. A comparative transcriptome and weighted gene co-expression network analysis (WGCNA) method was used to identifies hub genes positively associated with resistance to *A. flavus* in genotypes resistant (J 11, R) and susceptible (Zhonghua 12, S) and revealed 18 genes in *A. flavus* defenses [10].

Numerous strategies have been employed to prevent aflatoxin contamination in peanuts, including use of biological or chemical control agents [11,12], field measures prior to sowing, and cultivation of varieties with resistance to *A. flavus* infection and/or toxin production. Breeding and employment of resistant peanut varieties has been well recognized as an ideal approach for controlling the contamination risk. Progress has been achieved in genetic enhancement for aflatoxin resistance in peanut worldwide. RNAi was used to silence the aflatoxin producing genes in fungi and achieved about a 90% reduction in aflatoxin content. Co-expression of antifungal defensins and hpRNAs targeting mycotoxin genes has been tried, and the toxin level in the transgenic peanut plants was much reduced [13]. Sharma et al. cultivated a peanut germplasm with high level resistance to *A. flavus* infection and aflatoxin contamination by overexpressing antifungal plant defensins MsDef1 and MtDef4.2, and through host-induced gene silencing of aflM and aflP genes [14]. Metabolite analysis showed that pipecolic acid was the key component of peanut resistance to *A. flavus*, and exogenous treatment of susceptible peanut cultivars with pipecolic acid could reduce *A. flavus* infection in seeds [15]. More work has been done in peanut germplasm screening for resistance, and several resistant genotypes including J 11 [16], ICG 12625 [17], Xinhuixiaoli and AH 7223 [18] have been identified, and even used, in breeding. Unfortunately, these resistant lines have been rarely used in commercial production because of their low yield potentials, mostly due to possessing small pods and seeds. Integrating the aflatoxin resistance and large seed trait would be essential for developing high yield and aflatoxin resistant varieties.

In recent years, SNP markers have been developed for constructing high-density genetic linkage maps, and several QTLs for resistance to aflatoxin contamination have been identified. Liang et al. identified six QTLs associated with *A. flavus* infection by using SSR linkage maps [19]. Zhang et al. identified one major QTL for resistance to aflatoxin accumulation in maize by genome-wide association analysis (GWAS) and traditional linkage mapping analysis [20]. Yu et al. used a recombinant inbred line (RIL) population from a cross combination between ICG 12625 and Zhonghua 10 for QTL mapping and identified one major QTL for resistance to *A. flavus* infection on chromosome A10, and two major QTLs for resistance to aflatoxin production (total content of AFB_1_ and AFB_2_) on chromosomes A 03 and A10 [21]. By using a RIL population (Xinhuixiaoli × Yueyou 92), Khan et al. identified two QTLs for *A. flavus* infection resistance with 4.4–18.11% PVE on A03 and B04 [22]. J 11 is a well-known peanut line with *A. flavus* infection resistance and the resistance has been repeatedly confirmed in different research groups in the world. Moreover, Ramon et al. inoculated J 11 with *A. flavus* suspension and found this genotype also had resistance to aflatoxin production [23]. In our previous study, we identified six QTLs for *A. flavus* infection resistance in J 11 by phenotyping the percent seed infection index (PSII) and found that pyramiding all the six QTLs could significantly enhance the infection resistance [24]. However, the major loci associated with the aflatoxin production resistance in J 11 are unknown.

In this study, a RIL population with J 11 being its resistant parent was used to map major QTLs for AP resistance by phenotyping toxin content under artificial inoculation and for hundred-seed weight (HSW). Furthermore, the relationship between the aflatoxin resistance and seed weight with the corresponding combinations of major QTL loci for AP, PSII and HSW was analyzed and elite segregant lines with improved aflatoxin resistance and yield potential were selected from the RILs.

## 2. Materials and Methods

### 2.1. Plant Materials

A peanut recombined inbred lines (RILs) population consisting of 200 lines was derived from a cross combination between Zhonghua 16 and J 11. The female parent of the RIL, Zhonghua 16, was a peanut variety susceptible to *A. flavus* infection with high yield potential developed by the Oil Crops Research Institute of Chinese Academy of Agricultural Sciences (OCRI-CAAS). The male parent of the RIL, J 11, was a peanut genotype resistant to *A. flavus* infection introduced from the International Crops Research Institute for the Semi-Arid Tropics (ICRISAT), Hyderabad, India. For three consecutive years (2017, 2018 and 2019), the RILs of three generations (F_7_–F_9_) were planted in the experimental station of OCRI-CAAS in Wuhan, China. The field trials were conducted following a method described by Yu et al. [21], with the block design being completely randomized with three replications. After harvesting, the peanut pods were dried immediately. Healthy and mature seeds were selected for further investigation.

### 2.2. Phenotyping of RILs for Aflatoxin Production Resistance

The *A. flavus* strain AF2202 with strong capacity of seed invasion, colonization and aflatoxin production was used in artificial inoculation for phenotyping aflatoxin resistance. The methods for quantifying *A. flavus* infection and toxin content were as described by Yu et al. [21]. Fifteen seeds from each entry were selected for disinfection and rinsing, and then inoculated with 1 mL (2 × 10^6^ conidia/mL) of conidia suspension. The seeds were randomly and evenly placed in a sterile Petri dish, and 1 mL of conidia suspension was inoculated. The time of disinfection and cleaning was controlled within 13 min to ensure the consistency of water absorption of different materials. After inoculation, all Petri dishes were incubated in an incubator for 7 days, with a relative humidity of 85%, an air temperature of 30 °C, and a dark environment. After 7 days of inoculation, the spores on the surface of peanut kernels were washed with alcohol, dried and ground into fine powder, and 1 g powder was transferred to a 15 mL centrifuge tube medium, mixed with 5 mL of methanol and 1 mL of petroleum ether, and shaken at 190 rpm for 30 min on a shaker. After fully mixing, it was put into a centrifuge tube in a centrifuge at 3000 rpm for 6 min, 500 μL of the middle layer of methanol was put into a 15 mL centrifuge tube, and then diluted 20 times with 55% methanol. Aflatoxin content (ATC) (AFB_1_ and AFB_2_) in peanut kernel was quantified using a high-performance liquid chromatograph (Agilent Technologies, Santa Clara, CA, USA, 1200 series equipped with HPLC C18 4.6 mm × 250 mm, 5 nm column). The column was maintained at 30 °C in the system column heater. The mobile phase consisted of a methanol/water (45:55) mixture at a flow rate of 0.7 mL/min. The injection volume was 10 microliters, and the injection time was 17 min. Aflatoxin standard solution (CRM46304, Sigma-Aldrich, Munich, Germany) was used to establish a standard curve.

### 2.3. Statistical Analysis

Phenotypic data for each RIL and the parents were used for analysis of variance (ANOVA), means, broad-sense heritability, and correlation coefficient. Phenotypic data were analyzed using SPSS Statistics 22.0 statistical software. The broad-sense heritability of toxin content was calculated as *H^2^* = $\sigma_{g}^{2}$/($\sigma_{g}^{2}$ +$\sigma_{gXe}^{2}$/n + $\sigma_{e}^{2}$/rn), where $\sigma_{g}^{2}$ is the genetic variance component, $\sigma_{gXe}^{2}$ is the genotype-environment interaction variance component, $\sigma_{e}^{2}$ is the residual (error) variance component, and n and r were defined as the number of environments and the number of replicates, respectively.

The genetic linkage maps of 200 RILs including 2802 bin blocks with a total bin-map distance of 1573.85 cM was previously constructed in this laboratory [24].

### 2.4. QTL Analysis

QTL Cartographer 2.5 software was used to detect each trait in each environment via a composite interval mapping model in a high-density bin genetic map. The walk speed was set at 1 cM, and the logarithm of the odds (LOD) threshold was set at 2.5 to detect additive QTLs. The QTLs were named using an abbreviated trait name and corresponding chromosome number.

### 2.5. Conditional QTL Analysis

Conditional analysis was performed using Windows QTL Cartographer 2.5 software based on conditional phenotypic values y(ATC|HSW), y(ATC|PSII) and y(PSII|HSW), which were calculated by the mixed-model method using QGA Station 2.0 software (http://ibi.zju.edu.cn/software/qga/v2.0/) (accessed on 11 June 2022).

## 3. Results

### 3.1. Variation of Aflatoxin Production Resistance in the RILs

**Figure 1:**
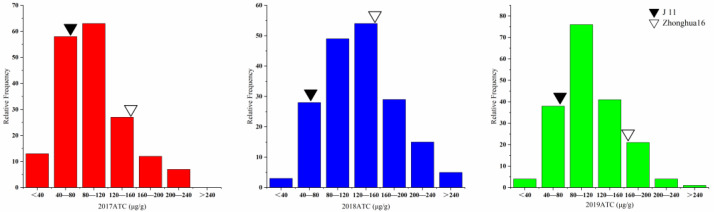
Phenotypic distribution of ATC in the RIL population with their parents in 3 years.

The aflatoxin contents of the two parents and the RILs were determined by artificial inoculation with a toxicogenic *A. flavus* strain in laboratory for three consecutive years (2017–2019). Significant differences in toxin content between Zhonghua 16 and J 11 were observed. The toxin content of Zhonghua 16 ranged from 159.01 to 164.07 μg/g, whereas J 11 ranged from 65.91 to 74.76 μg/g in the three environments. The toxin content of the RILs varied from 6.16 to 277.92 μg/g, 34.82 to 336.77 μg/g and 26.52 to 340.29 μg/g in the three years (Table 1). The value of toxin content in the RILs showed continuous variation with a bidirectional transgressed segregation (Figure 1). Based on integrated analysis of multiple environmental data, 15 lines were found to exhibit superiority over their parents in resistance to aflatoxin production. The distribution frequency was close to normal distribution. The correlation coefficient of the toxin contents across the multiple years ranged from 0.332 to 0.401 (Table S1). The results of an ANOVA for toxin content showed significant differences among genotypes, environments and genotypes × environments interactions at *p* < 0.001 (Table 2). The broad-sense heritability of the toxin content was estimated to be 0.659, indicating that the AP resistance was mainly controlled by genetic factors.

### 3.2. Correlation Analysis of ATC with HSW and SPII

**Figure 2:**
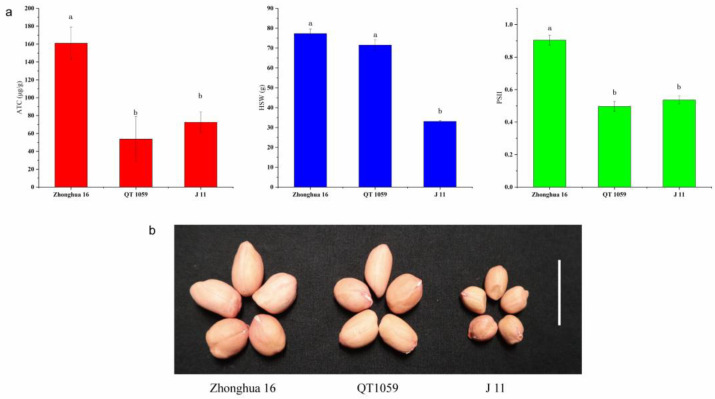
Phenotypic characterization of seeds from ‘Zhonghua 16’, ‘QT 1059’ and ‘J 11’. (**a**) Comparisons of ATC, HSW and PSII among ‘Zhonghua 16’, ‘QT 1059’ and ’J 11’. Histogram represented mean ± s.d. (*n* = 9 for ATC and PSII, *n* = 3 for HSW). Different letters in the graph mean the values are statistically different at *p* < 0.05 based on LSD mupltiple comparison. (**b**) Seed morphology of ‘Zhonghua 16’, ‘QT 1059’ and ‘J 11’. Scale bar: 2 cm. ATC aflatoxin content, HSW hundred seed weight, PSII percent seed infection index.

The correlation coefficients of ATC and HSW between the two years were 0.262 and 0.362 (Table S2). The correlation coefficients between ATC and SPII were 0.055 and 0.023 in 2017 and 2018, respectively, but 0.397 in 2019 (Table S3). These results suggest that the toxin content might be related to HSW and SPII to some extent. Although a positive correlation between ATC and HSW was found, the correlation coefficient was low, indicating that there might be some recombined lines possessing resistance to aflatoxin production with high HSW. There were 61, 16 and 36 lines in which the toxin contents were lower than the resistant parent J 11 in 2017, 2018 and 2019, respectively (Table S4). Among the resistant RILs, 13 possessed low HSW (less than 60 g). For the remaining two lines, HSW of QT-1089 was 63.12 g and 70.90 g in 2017 and 2018, respectively, and that of QT-1059 was 70.17 g and 74.50 g in 2017 and 2018, respectively. The ATC of QT-1059 was not significantly different from J 11, and the HSW of QT-1059 was not significantly different from Zhonghua 16. We also collected the PSII data of the 15 resistant lines, of which 13 were susceptible to *A. flavus* infection, and in the remaining two lines only QT-1059 showed stable resistance to *A. flavus* infection and its PSII was not significantly different from J 11 (Table 3, Figure 2).

### 3.3. QTLs for AP Resistance and HSW in the RILs

A genome-wide QTL analysis was conducted using high-density genetic maps constructed in a previous study [25] and the phenotypic data of the toxin content from the 200 RILs in three consecutive years in Wuhan. A total of 11 additive QTLs were identified with 4.61–11.42% PVE (Table 4). In 2017, 2018 and 2019 trials, five, five and four QTLs were identified, and they totally explained 32.33, 39.98 and 26.08% PVE, respectively. Their LOD values ranged from 2.65 to 6.58. Three QTLs were detected on A05 and B06, two were detected on B05 and B09, and one was detected on A08. The QTL *qAFTA05.1* was consistently detected in three years, showing 5.99–11.42% PVE. *qAFTB06.3* was repeatedly detected in two years with 8.23–10.63% PVE. The major QTL *qAFTB05.2* was only detected in one year with 9.90% PVE.

For HSW, in a previous study, an SSR map was used for QTL analysis. In this study, the phenotypic data were used for QTL analysis by SNP map, and six QTLs were detected on five chromosomes (A05, A08, A10, B01 and B06) with a range of 3.99% to 29.02% PVE (Table 4), including three major QTLs. Their LOD values ranged from 3.16 to 20.69. Major QTLs, namely *qHSWA05*, *qHSWA08* and *qHSWB06*, were consistently detected in two environments and showed 28.34–29.02%, 5.20–9.88% and 4.76–10.46% PVE, respectively. The main QTLs detected by SNP map and SSR map were co-located (Table S5). Favorable alleles of QTLs for HSW were all from Zhonghua 16.

**Figure 3:**
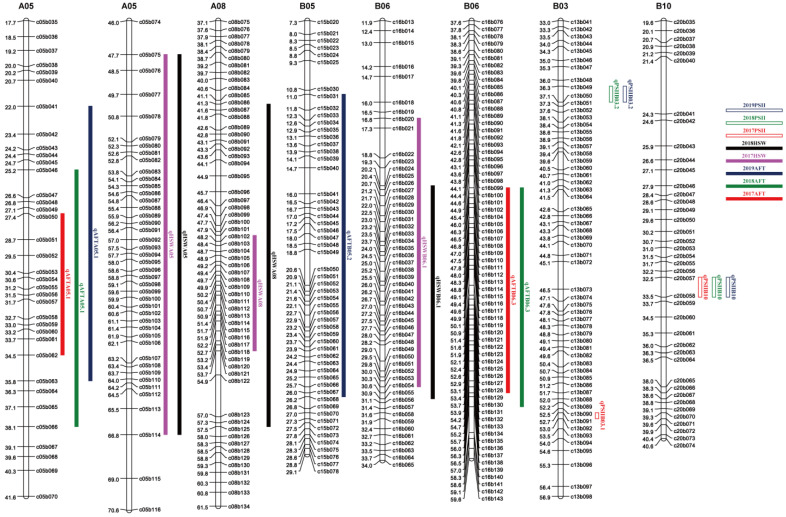
Distribution of QTLs for traits of resistance to aflatoxin contamination and HSW on the genetic map.

The major QTLs of ATC and HSW were located on chromosomes A05 and B06, but the physical locations of these loci did not overlap. In a previous study, we identified six QTLs related to PSII and found that these six QTLs were not on the same chromosome as the QTLs of ATC (Figure 3).

### 3.4. Conditional QTL Mapping

Since several QTLs for ATC and HSW were found to be co-located on the same chromosomes, suggesting that genetic relationship between these two traits might exist. In order to investigate the interactions between ATC and HSW at QTL level, conditional QTL analysis was conducted using conditional phenotypic values y(ATC|HSW). The mean of values for ATC and HSW across multiple environments were used to calculate y(ATC|HSW). Six conditional QTLs with 4.42–12.86% PVE were identified (Table 5), including one additional QTL for toxin content. However, two QTLs were missing when compared with unconditional QTL analysis, including major QTL *qAFTB06.3* for toxin content (Table 5). The result shows that there was little influence on identification of QTLs for HSW when QTL analyses conditioned on toxin content.

Conditional QTL analysis was performed with conditional phenotypic values y(ATC|PSII). The mean of values for ATC and PSII across multiple environments were used to calculate y(ATC|PSII). Seven QTLs for toxin content were identified in unconditional analysis, whereas two of them failed to be detected when ATC was conditioned on PSII (Table 5). The major QTL *qAFTB05.2* was not found in conditional mapping. Another major QTL *qAFTA05.1* obviously decreased the additive effect (7.72% PVE) compared to that of the unconditional QTL (13.96% PVE) (Table 5). The additive effect of the major QTL *qAFTB06.3* (14.83% PVE) was significantly higher than that of the unconditional QTL (5.15% PVE) (Table 5).

We also used conditional phenotypic value y (PSII | HSW) for conditional QTL analysis.

One additional QTL (*qPSIIB01*) was identified with 4.99% PVE in conditional QTL analysis (Table 5). Of the four QTLs for PSII identified in unconditional mapping, one was not detected when PSII was conditioned on HSW (Table 5). The other three QTLs (*qPSIIB03.1*, *qPSIIB03.2* and *qPSIIB10*) slightly enhanced the additive effect (4.94, 5.77 and 9.48% PVE) compared to that of the unconditional QTLs (6.05, 6.02 and 10.31% PVE) (Table 5).

Overall, HSW exhibited significant suppression on expression of major QTL (*qAFTB06.3*) for ATC, PSII significantly inhibited the expression of major QTL *qAFTA05.1* but enhanced the expression of major QTL *qAFTB06.3* for toxin content. *qPSIIA08* was severely affected by HSW. These results suggest that ATC, HSW and PSII can influence each other to some extent.

### 3.5. Phenotypic Effect of Pyramiding the Major QTLs

Favorable alleles of *qAFTA05.1* and *qAFTB06.3* were from J 11, while favorable alleles of *qAFTB05.2* were from Zhonghua 16. “AA”, “BB” and “CC” represented paternal genotype (Zhonghua 16) for *qAFTA05.1*, *qAFTB06.3* and *qAFTB05.2*, respectively, and maternal genotype (J 11) were designated as “aa”, “bb” and “cc”, respectively. In the RILs, genotype “AABBcc” (135.61, 184.50 and 145.34 μg/g) accumulated significantly higher aflatoxin in all environments compared to other genotypes (AABBCC, AAbbcc, aaBBcc, aaBBcc, AAbbCC, aaBBCC, aabbcc and aabbCC) (Table 6), indicating that combination of one or more resistant alleles of three major QTLs (*qAFTA05.1*, *qAFTB06.3* and *qAFTB05.2*) could significantly decrease toxin content. In addition, the lines with the genotype “aabbCC” accumulated 70.96 μg/g and 94.81 μg/g toxin in 2017 and 2019, which were significantly lower than that of lines with the genotype of the resistant parent J 11 (aabbcc). Fifteen lines exhibiting superiority over resistant parent J 11 in toxin content, and all the lines harbored at least two resistant alleles of major QTLs. All the results demonstrate that pyramiding favorable alleles of major QTLs could decrease aflatoxin production.

For HSW, the genotype with *qHSWA05*, *qHSWA08 and qHSWB06* derived from Zhonghua 16 were designated as “DD”, “EE” and “FF”, while the genotypes from J 11 were designated as “dd”, “ee” and “ff”, respectively. HSW of “DDEEFF” genotypes (68.85 g and 73.55 g) were significantly higher than that of “ddeeff” genotypes in two consecutive years in Wuhan (Table 6). The result indicates that combination of the *qHSWA05*, *qHSWA08* and *qHSWB06* can enhance the HSW in peanut.

In analysis of conditional QTL, we found that the major QTL *qAFTB06.3* was affected by both HSW and PSII, so we selected the major QTLs *qAFTA05.1* and *qAFTB05.2* for aggregation. Similarly, QTLs for HSW (*qHSWA05*, *qHSWA08* and *qHSWB06*) and PSII (*qPSIIB03.1*, *qPSIIB03.2* and *qPSIIB10*) were selected for polymerization of multiple traits.

Among the 200 RILs, QT 1059 exhibited lower ATC and similar PSII compared with the resistant parent J 11, and HSW comparable to the high-yielding parent Zhonghua 16. This special RIL simultaneously harbored favorable alleles of HSW (*qHSWA05*, *qHSWA08* and *qHSWB06*), two resistant alleles of AP resistance (*qAFTA05.1* and *qAFTB05.2*), and three resistant alleles of PSII (*qPSIIB03.1*, *qPSIIB03.2* and *qPSIIB10*). Integration of these favorable alleles was the reason for its elite phenotypic traits.

## 4. Discussions

Aflatoxin contamination in peanuts and peanut products is a great concern to food safety globally. *Aspergillus* fungal infection and consequent aflatoxin contamination can occur in pre-harvest and post-harvest stages in peanut production and utilization. Breeding and planting resistant peanut varieties is the most effective practical approach for managing the contamination. Mixon and Rogers were the first to suggest the use of resistant varieties to curb the problem in peanut [26]. However, the genetic enhancement for resistance to *A. flavus* infection and toxin formation has progressed slowly, largely due to the productivity or yield potential of most improved resistant varieties are generally lower than the susceptible ones, for which lack of efficient molecular markers for aflatoxin resistance and yield components in breeding has been an important reason. Moreover, the relationship between aflatoxin resistance and seed size has not been well investigated at QTL level. In a previous study on resistance to aflatoxin production, Yu et al. [21] used multi-environmental phenotypical data to map QTLs for resistance to production of aflatoxin B_1_ and aflatoxin B_2_ in ICG 12625, from which, 12 QTLs were identified, and *qAFB1B06.1* and *qAFB1A07* were co-localized with *qAFB2B06* and *qAFB2A07* on LG B06 and B07, respectively. What is more, a strong interaction between resistance to production of AFB_1_ and AFB_2_ was confirmed by conditional QTL mapping. In this study, we quantified the production of aflatoxin B_1_ and aflatoxin B_2_ and performed QTL analysis on the total toxin content, from which 11 QTLs were detected including three major QTLs on chromosomes A05, B05 and B06. Compared to the study of Yu et al. [21], the QTLs on chromosomes A08 and B09 were newly identified in this study, indicating that genetic differences between ICG 12625 and J 11 might exist for genetic basis for resistance to aflatoxin formation.

In this study, six QTLs related to HSW were identified, including three major ones on chromosomes A05, A08 and B06. There have been several reports on QTL identification for yield-related traits, such as seed weight and size. Zeng et al. identified 16 QTLs for HSW by SSR, and two major QTLs were mapped on chromosome A05 and B06 [27]. We detected a new major loci *qHSWA08*, which may be related to the use of a SNP genetic linkage map. Luo et al. identified a major QTL related to HSW on A05 with physical coverage of 99.5 Mb–99.78 Mb [28], which was overlapped with the major QTL *qHSWA05* (100.1-113 Mb). Huang et al. reported a QTL *qHSWA8* for HSW on A08 [29], but the physical position of *qHSWA8* is unknown. Zhang et al. [30] and Chen et al. [31] identified QTLs on B06 with physical coverage of 7.33 Mb–21.71 Mb and 10.6 Mb–21.6 Mb, respectively, and they were co-localized with the major QTL *qHSWB06* (7.12–40.77 Mb) detected in this study.

Until now, the relationship between resistance to *A. flavus* infection or toxin formation with yield-related traits in peanut has been rarely addressed. We conducted a phenotypic correlation analysis and found that the correlation between HSW and aflatoxin content under in vitro inoculation in 2017 was 0.262 and 0.322 in 2018. In previous research, Ding et al. found that aflatoxin contents were positively correlated with pod or seed size [32]. In this study, we found that *qHSWA05* for HSW was co-localized with *qAFTA05.2* and *qAFTA05.3* for toxin content. In order to reveal the relationship between toxin content and HSW, we conducted conditional QTL analysis for ATC and HSW. When ATC was conditioned on HSW, the two major QTLs (*qAFTA05.1* and *qAFTB05.2*) for ATC were not suppressed, and *qAFTB06.3* failed to be detected. Similarly, conditional QTL analysis was also performed for PSII and HSW. When PSII was conditioned on HSW, *qPSIIA08* could not be identified, but *qPSIIB03.1*, *qPSIIB03.2* and *qPSIIB10* were still present with slightly enhanced PVE (Table 5). These results showed that HSW of peanut had a slight effect on resistance to *A. flavus* infection, and they might share the same or similar regulation pathways. We also analyzed the relationship between resistance to ATC and *A. flavus* infection, and a significant correlation between AP resistance and *A. flavus* infection resistance was observed except for in 2019 (the correlation coefficient was 0.397, Table S3). In a previous study, six QTLs related to PSII were identified on chromosomes A05, A08, B01, B03, and B10 [18]. We also detected two QTLs linked to ATC on chromosomes A05 and A08, and the physical coverage of *qPSIIA05* and *qPSIIA08* overlapped with *qAFTA08* and *qAFTA05.2*. Conditional QTL analysis showed that PSII had a slight effect on ATC. The results were similar to those in previous reports [33], indicating that the resistance of peanut to *A. flavus* infection and to toxin formation might have a similar regulatory pathway, and their regulation was not completely independent. Therefore, pyramiding major QTLs for ATC (*qAFTA05.1* and *qAFTB05.2*), HSW (*qHSWA05*, *qHSWA08 and qHSWB06*) and PSII (*qPSIIB03.1*, *qPSIIB03.2* and *qPSIIB10*) could simultaneously enhance resistance and yield potential.

Interestingly, we found that a segregated variant line from the RILs, QT 1059, with desirable aflatoxin resistance and high HSW. QT 1059 simultaneously possessed the resistant alleles for ATC (*qAFTA05.1*, *qAFTB05.2*), PSII (*qPSIIB03.1*, *qPSIIB03.2* and *qPSIIB10*) and three favorable alleles of HSW (*qHSWA05*, *qHSWA08* and *qHSWB06*). Compared with the resistant parent J 11, QT 1059 had reduced ATC (17 μg/g), PSII (0.0397) and increased HSW (72.33 g). The value of HSW in QT 1059 also approached that in the high-yield parent Zhonghua 16. These results might indicate that combination of the major QTLs for ATC (*qAFTA05.1* and *qAFTB05.2*) and HSW (*qHSWA05*, *qHSWA08* and *qHSWB06*) could improve aflatoxin resistance and yield potential of peanut concurrently.

## 5. Conclusions

From combined analysis of the phenotypic data of the RILs derived from Zhonghua 16 × J 11 and the previously constructed high density genetic map of the same RILs, eleven QTLs associated with aflatoxin production resistance were detected in three environments. Six QTLs associated with HSW were also detected in two environments. A combination of favorable alleles of major QTLs for ATC (*qAFTA05.1* and *qAFTB05.2*), PSII (*qPSIIB03.1*, *qPSIIB03.2* and *qPSIIB10*) and HSW (*qHSWA05*, *qHSWA08* and *qHSWB06*) could significantly enhance aflatoxin resistance and yield potential. A special line from the RIL, QT-1059, was identified with enhanced aflatoxin resistance and high HSW. The results obtained in this study provide a meaningful foundation for peanut breeding in terms of coordinated enhancement of both resistance and productivity.

## Figures and Tables

**Table 1:** The phenotypic variation of ATC in the RIL population.

Env	Parents		RILs		CV
	Zhonghua 16	J 11	Range	Mean ± SD	
2017	159.01 ± 21.00	73.64 ± 11.55 **	6.16–277.92	98.04 ± 53.95	0.55
2018	158.98 ± 4.43	65.91 ± 8.90 **	34.82–336.77	134.33 ± 69.62	0.52
2019	164.07 ± 21.09	74.76 ± 12.85 **	26.52–340.29	112.16 ± 58.88	0.52

Env, environment; SD, standard deviation; CV, coefficient of variation; ** Difference is significant at *p* < 0.01 level between parents.

**Table 2:** Analysis of variance for the ATC in the RIL population across three environments.

Source	*df*	Mean Square	*F*-Value	*p*-Value	*H* ^2^
Genotypes	184	11,832.21	5.05	<0.001	0.692
Environments	2	161,839.88	69.13	<0.001	
Genotypes × Environments	361	3649.54	1.56	<0.001	
Error	1048	2341.05			

**Table 3:** Detailed ATC, HSW and PSII of 15 accessions and parents across different environments.

Line	2017ATC (μg/g)	2018ATC (μg/g)	2019ATC (μg/g)	2017HSW (g)	2018HSW (g)	2017PSII	2018PSII	2019PSII
QT 1026	37.19	61.73	67.91	57.36	58.81	0.8866	0.7849	0.7094
QT 1029	53.30	40.32	67.67	54.96	55.78	0.9163	0.7802	0.6336
QT 1041	26.17	47.91	39.11	46.37	54.27	0.9889	0.6795	0.5491
QT 1044	76.83	63.22	62.99	58.84	53.70	0.7778	0.6784	0.6167
QT 1059	37.27	56.98	67.15	70.17	74.50	0.4727	0.5300	0.4866
QT 1068		71.53	38.88	52.74	51.88	0.3847	0.7384	0.3492
QT 1088	42.04	68.60	85.92	58.09	58.61	0.5134	0.8018	0.5640
QT 1089	29.11	34.82	73.43	63.12	70.90	0.9147	0.9814	0.8778
QT 1105	37.65	73.31	76.81	58.19	57.30	0.9037	0.8651	0.7982
QT 1120	41.41	77.67	75.75	41.70	48.61	0.8105	0.7684	0.6917
QT 1126	54.04	66.12	78.08	46.88	51.59	0.9423	0.6647	0.6291
QT 1137	51.24	77.02	46.87	49.46	52.97	0.6303	0.6592	0.4447
QT 1141	49.38	75.10	38.99	41.88	46.68	0.7683	0.7592	0.4219
QT 1142	32.75	66.52	77.88	50.52	48.42	0.9356	0.7575	0.8360
QT 1166	63.48	45.20	56.68	45.19	48.44	0.8136	0.7474	0.6883
Zhonghua 16	164.07	159.01	158.98	79.27	77.90	0.8704	0.9115	0.9300
J 11	74.76	73.64	65.91	32.49	33.67	0.5597	0.5383	0.5104

ATC, aflatoxin content; HSW, hundred seed weight; PSII, percent seed infection index.

**Table 4:** Additive QTLs for resistance to ATC and HSW in the RIL population across three and two environments.

Trait	QTL	LG	Env	CI (cM)	Marker Interval	LOD	PVE(%)	Add
ATC	***qAFTA05.1***	**A05**	**2017**	**27.41–35.52**	**c05b050–c05b062**	**4.44**	**7.95**	**12.77**
			**2018**	**25.19–38.08**	**c05b046–c05b066**	**6.35**	**11.42**	**18.09**
			**2019**	**22.02–35.80**	**c05b041–c05b063**	**3.29**	**5.99**	**10.64**
	*qAFTA05.2*	A05	2017	57.49–59.07	c05b093–c05b098	3.12	5.49	11.06
	*qAFTA05.3*	A05	2017	63.71–70.01	c05b109–c05b115	3.11	5.83	11.07
	*qAFTA08*	A08	2019	25.15–25.91	c08b048–c08b050	2.82	4.99	−9.79
	*qAFTB05.1*	B05	2019	8.30–8.55	c15b022–c15b023	2.73	5.2	−9.91
	*qAFTB05.2*	B05	2019	11.05–26.25	c15b031–c15b068	5.37	9.9	−13.79
	*qAFTB06.1*	B06	2017	39.06–39.56	c16b081–c16b083	2.65	4.83	9.95
	*qAFTB06.2*	B06	2018	39.81–44.65	c16b084–c16b101	4.54	7.52	14.86
	***qAFTB06.3***	**B06**	**2017**	**46.04–56.26**	**c16b105–c16b137**	**4.64**	**8.23**	**12.91**
			**2018**	**46.04–57.03**	**c16b105–c16b139**	**6.58**	**10.63**	**17.6**
	*qAFTB09.1*	B09	2018	39.57–41.65	c19b079–c19b086	2.86	4.61	11.58
	*qAFTB09.2*	B09	2018	47.11–49.96	c19b104–c19b112	3.63	5.8	12.93
HSW	***qHSWA05***	**A05**	**2017**	**47.7–68.86**	**c05b075–c05b114**	**17.43**	**28.34**	**5.61**
			**2018**	**47.7–68.86**	**c05b075–c05b114**	**20.69**	**29.02**	**5.75**
	***qHSWA08***	**A08**	**2017**	**47.94–53.69**	**c08b101–c08b121**	**3.86**	**5.2**	**2.38**
			**2018**	**41.34–57.53**	**c08b086–c08b125**	**8.39**	**9.88**	**3.36**
	*qHSWA10.1*	A10	2018	5.22–9.68	c10b006–c10b011	3.58	3.99	2.11
	*qHSWA10.2*	A10	2017	18.53–27.98	c10b021–c10b051	3.84	5.16	2.37
	*qHSWB01*	B01	2018	43.54–49.48	c11b077–c11b095	3.16	3.49	1.97
	***qHSWB06***	**B06**	**2017**	**16.51–20.17**	**c16b019–c16b024**	**3.55**	**4.76**	**2.29**
			**2018**	**19.31–39.81**	**c16b023–c16b084**	**8.76**	**10.46**	**3.42**

ATC, aflatoxin content; HSW, hundred seed weight; LG, linkage group; Env, Environment; Cl, confidence interval of QTLs; PVE, phenotypic variance explained; Add, additive effect. QTLs identified in more than one environment are highlighted in bold.

**Table 5:** Comparison of unconditional and conditional QTLs for ATC and HSW in the RIL population.

Condition	QTL	Marker Interval	Unconditional QTL PVE (%)	Conditional QTL PVE (%)
ATC|HSW	*qAFTA05.1*	c05b040–c05b069	13.96	12.86
	*qAFTA05.3*	c05b091–c05b115	9.19	
	*qAFTA08*	c08b049–c08b050	4.99	4.42
	*qAFTB05.1*	c15b018–c15b024		7.65
	*qAFTB05.2*	c15b031–c15b063	5.88	10.15
	*qAFTB06.3*	c16b111–c16b114	5.15	
	*qAFTB09.1*	c19b080–c19b090	4.05	6.42
	*qAFTB09.2*	c19b111–c19b112	4.11	6.97
ATC|PSII	*qAFTA05.1*	c05b040–c05b069	13.96	7.72
	*qAFTA05.3*	c05b091–c05b115	9.19	7.47
	*qAFTA08*	c08b049–c08b050	4.99	
	*qAFTB05.2*	c15b031–c15b063	5.88	
	*qAFTB06.3*	c16b111–c16b114	5.15	14.83
	*qAFTB09.1*	c19b080–c19b090	4.05	4.52
	*qAFTB09.2*	c19b111–c19b112	4.11	4.75
PSII|HSW	*qPSIIA08*	c08b096–c08b137	9.58	
	*qPSIIB01*	c11b115–c11b116		4.99
	*qPSIIB03.1*	c13b066–c13b069	4.94	6.05
	*qPSIIB03.2*	c13b090–c13b092	5.77	6.02
	*qPSIIB10*	c20b046–c20b064	9.48	10.31

ATC, aflatoxin content; HSW, hundred seed weight, PSII, percent seed infection index.

**Table 6:** Phenotypic effect of major QTLs for ATC (*qAFTA05.1*, *qAFTB06.3* and *qAFTB05.2*) and HSW (*qHSWA05*, *qHSWA08* and *qHSWB06*) in the RIL population.

Trait	Genotype	2017	2018	2019
ATC (μg/g)	AABBcc	135.61 ± 64.80 ^a^	184.50 ± 81.90 ^a^	145.34 ± 81.26 ^a^
	AABBCC	115.56 ± 62.33 ^b^	157.81 ± 67.57 ^b^	113.65 ± 57.45 ^bc^
	AAbbcc	114.62 ± 50.09 ^bc^	141.69 ± 61.60 ^bc^	140.12 ± 63.22 ^a^
	aaBBcc	97.25 ± 46.33 ^cd^	147.00 ± 72.43 ^b^	115.88 ± 56.94 ^bc^
	AAbbCC	95.83 ± 48.95 ^cd^	114.76 ± 63.41 ^cd^	116.32 ± 54.68 ^bc^
	aaBBCC	91.55 ± 48.41 ^de^	115.78 ± 52.82 ^cd^	94.70 ± 47.70 ^c^
	aabbcc	76.21 ± 33.91 ^ef^	97.94 ± 54.73 ^d^	117.51 ± 52.53 ^bc^
	aabbCC	70.96 ± 38.22 ^f^	111.09 ± 59.90 ^cd^	94.81 ± 48.12 ^c^
HSW (g)	DDEEFF	68.85 ± 10.56 ^a^	73.55 ± 7.43 ^a^	-
	DDEEff	64.06 ± 8.39 ^ab^	66.67 ± 8.81 ^b^	-
	DDeeFF	59.82 ± 7.71 ^bc^	66.02 ± 7.69 ^b^	-
	DDeeff	57.41 ± 6.39 ^c^	59.86 ± 6.71 ^c^	-
	ddEEFF	58.57 ± 5.58 ^bc^	60.00 ± 6.41 ^c^	-
	ddEEff	51.41 ± 7.22 ^d^	55.21 ± 8.46 ^c^	-
	ddeeFF	51.70 ± 8.77 ^d^	55.80 ± 5.92 ^c^	-
	ddeeff	46.66 ± 6.46 ^d^	49.21 ± 2.33 ^d^	-

ATC, aflatoxin content; HSW, hundred seed weight; In each column, the values followed by different letters mean statistically different at *p* < 0.05 based on LSD multiple comparison.

## Data Availability

Not applicable.

## Supplementary Materials

The following supporting information can be downloaded at: https://www.mdpi.com/article/10.3390/genes14030625/s1, Table S1: Correlation analysis of ATC in RIL population. Table S2: Correlation analysis of aflatoxins contents and yield-related trait in RIL population. Table S3: Correlation analysis of aflatoxins contents and PSII in RIL population. Table S4: Integrated analysis of ATC and HSW in the RILs. Table S5: Comparison of QTLs detected by SNP map and SSR map.
